# Spatial patterning of self-harm rates within urban areas

**DOI:** 10.1007/s00127-018-1601-3

**Published:** 2018-09-26

**Authors:** Catherine Polling, Ioannis Bakolis, Matthew Hotopf, Stephani L. Hatch

**Affiliations:** 1 Department of Psychological Medicine, Institute of Psychiatry, Psychology and Neuroscience, King’s College London, London, UK; 2 South London and Maudsley NHS Foundation Trust, London, UK; 3 Department of Biostatistics and Health Informatics, Institute of Psychiatry, Psychology and Neuroscience, King’s College London, London, UK

**Keywords:** Self-harm, Urban health, Small-area analysis, Deprivation, Ethnicity

## Abstract

**Purpose:**

Urban areas are usually found to have higher rates of self-harm, with deprivation the strongest predictor at area-level. We use a disease mapping approach to examine how self-harm is patterned within an urban area and its associations with deprivation, urbanness and ethnicity.

**Methods:**

Data from clinical records on individuals admitted for self-harm for 725 small areas in South East London were included. Bayesian hierarchical models explored the spatio-temporal patterns of self-harm admission rates and potential associations with proximity to city centre, population density, percentage greenspace and non-white ethnic-minority populations. All models were adjusted for area-level deprivation, social fragmentation and hospital of admission.

**Results:**

There were 8327 first admissions for self-harm during the study period. Self-harm admission rates varied fourfold across the study area, with lower rates close to the city centre [adjusted standardised admission ratio, closest versus furthest quartile 0.71(95% CrI 0.54–0.96)]. Deprivation was associated with self-harm but partially masked rather than explained the spatial pattern, which strengthened after adjustment. After adjustment for deprivation, hospital of admission and social fragmentation, greenspace, population density and ethnicity were not associated with self-harm rates.

**Conclusion:**

Proximity to the city centre was associated with lower rates of self-harm, but the usual operationalisations of urbanness, population density and greenspace, were not. Deprivation did not explain the spatial patterning, nor did ethnicity. While nationally self-harm rates are higher in urban and deprived areas, this cannot be extrapolated to mean that within cities the inner-city is the highest risk area nor that risk will be principally patterned according to deprivation.

**Electronic supplementary material:**

The online version of this article (10.1007/s00127-018-1601-3) contains supplementary material, which is available to authorized users.

## Introduction

Self-harm, through both self-poisoning and self-injury [1], represents a significant public health challenge in the UK, with evidence from administrative [2] and survey data [3] in England suggesting rates may have been rising since 2008. Managing self-harm has a large impact on health services with over 100,000 general hospital inpatient admissions [4] in England each year. For individuals, self-harm requiring medical attention represents mental distress and usually disorder [5], damage to physical health [1] and is the strongest single risk factor for future suicide [6].

Rates of self-harm vary substantially between areas and communities [7]. Understanding how and why this occurs can inform planning of preventative interventions and services as well as offering the potential to illuminate its more distal causes [8]. Ecological studies of self-harm have attempted to do this by looking at small area-level associations with rates of self-harm [7]. The strongest and most consistent association found is with deprivation [8–11]. Social fragmentation, operationalised using indicators of less settled and cohesive communities [12], has also been shown to be associated with self-harm, although less strongly than with suicide [13].

The current literature suggests a broad pattern of urban environments having higher rates of self-harm, contributed to by higher levels of both deprivation and social fragmentation, but not fully explained by either of these [8, 10, 11, 13, 14]. Research on rural/urban associations with self-harm has generally used broad categories [10, 11, 14, 15] so does not elucidate what aspects of urban environment are important for self-harm, and have not examined the role of ethnicity. It has been proposed that stressors related to urban living, for example exposure to noise, air pollution, crime and a poorer quality built environment, may be damaging to mental health [11].

There is some suggestion of variation within urban environments, for example Dublin has lower rates of self-harm than other Irish cities despite being the most urbanised [10], and one UK-wide study suggested rates may be highest in suburban areas and lower in the inner cities [9]. Likewise, suicide rates, which tend to follow trends in self-harm rates [2], have fallen in inner London, a change that is not explained by changes in employment or social fragmentation [16]. Such variation within urban areas remains unexplained. One hypothesis is that differences in ethnicity between populations may contribute to spatial patterning [16] as historically ethnic-minority populations in the UK have been concentrated in inner-city areas particularly inner London [17]. There is some evidence that rates of self-harm differ by ethnicity [18], but the nature of the association in the UK is unclear, with studies finding both higher [18–20] and lower [21] rates of self-harm in different minority groups when compared to the white British population.

## Aims

This study investigates small-area variations in rates of self-harm admission between 2007 and 2016 in a single urban area: South East London. We used a disease mapping approach allowing both associations and geographical patterning to be investigated. Our aims were to determine: (1) whether there is variation in general hospital inpatient admission rates after controlling for deprivation, social fragmentation and hospital admission practices; (2) whether such variation is spatially patterned (3) whether it is associated with urbanness, and (4) whether ethnicity explains any such association.

## Methods

### Study area

**Fig. 1:**
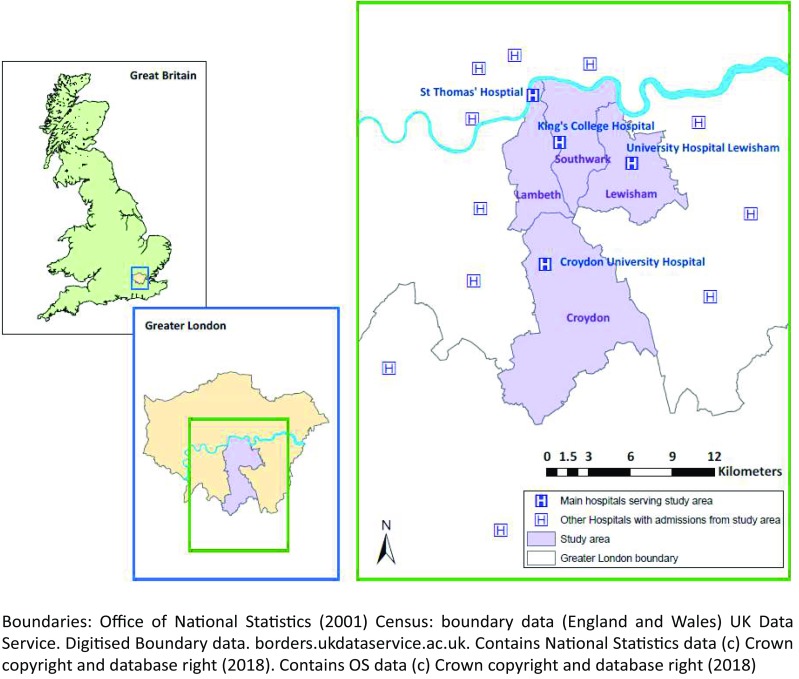
Location of study area

The study area, shown in Fig. 1, consists of four London boroughs: Lambeth, Southwark, Lewisham and Croydon, with a population of 1.2 million. The area stretches from central London to the border of the Greater London area, encompassing substantial variation in measures of urbanness as well as deprivation, social fragmentation and ethnicity [22] as shown in Table 1 and mapped in Supplementary Figure 1. We used a very local definition of area, 728 Lower Super Output Areas (LSOA, average population 1700), in order to capture the heterogeneity in area type across small distances within London, which may have been obscured in previous UK studies which have used larger administrative units [9, 11]. Secondary mental health care for the whole area is provided by South London and Maudsley NHS Foundation Trust (SLaM). By using an area served by one mental health care provider and adjusting for hospital of admission, we aimed to remove biases due to provider admission policies that may have affected previous studies using admission data [9].

**Table 1:** Characteristics of lower super output areas (LSOAs) within study area

	Mean	SD	Minimum	Maximum
Population per LSOA	1697	270	1043	3431
Households per LSOA	706	148	436	1784
Self-harm hospital admissions^a^	11.5	6.1	1	38
Deprivation				
IMD 2010 national centile^b^	68.0	18.8	4.3	97.6
Social fragmentation				
Congdon index^c^ average *z*-score	0.00	0.81	− 2.18	2.28
Urbanness				
Proximity to city centre (m)	9659	5051	1167	23,522
Population density (person/ha)	90.4	35.2	5.3	165.5
Greenspace (%)	0.19	0.16	0.00	0.91
Non-white ethnic minorities (%)	44.8	16.0	8.0	84.9

^a^First admission per individual counted

^b^Higher number denotes greater deprivation, centile within all areas in England and Wales

^c^Average of *z*-scores for percentage of: households privately renting; single person households; adults (over 16 years) unmarried and individuals living at a different address a year ago, UK census 2011

### Data

Data was accessed via the Clinical Records Interactive Search system (CRIS), a case register created from the anonymised electronic patient record of SLaM [23]. This is linked to Hospital Episode Statistics (HES), routine administrative data collected by all National Health Service (NHS) hospitals in the UK [24]. Population denominator data were taken from Office of National Statistics mid-year estimates [25]. Due to differences in the reporting geographies between HES and ONS data, six LSOAs had to be merged into 3 to make data comparable, hence 725 areas were used in analyses.

### Outcome

Admission for self-harm was defined as an individuals’ first episode of inpatient care in a general hospital with an ICD-10 diagnostic code in the range X60–X84 (Intentional self-harm) [26]. All episodes between 1/1/2007 and 31/3/2016 for individuals aged over 15 years resident in the study area were included, regardless of which hospital they were admitted to. To allow individual years to be compared, where admission rates and rate ratios by year were calculated an individuals’ first episode of inpatient care *each year* was included.

### Exposures

Deprivation was measured using the Index of Multiple Deprivation 2010 (IMD) [27], a composite measure summarising multiple dimensions of deprivation at LSOA level in England. Social fragmentation, an indicator of less settled and cohesive communities [12] which has also been shown to be associated with self-harm, was measured using the Congdon Index [12], created by combining *z*-scores for percentage of: households privately renting; single person households; adults (over 16 years) unmarried and percentage of individuals living at a different address a year ago, all taken from the UK census 2011. Ethnicity was measured using the percentage of the population from non-white ethnic groups from the UK census 2011.

Urbanness was measured as (1) LSOA centroid’s proximity to the city centre, defined as the conventional location, Charing Cross, with the furthest out LSOA set as 0; (2) population density within 1 km of the centroid of each LSOA, calculated using ArcGIS software; (3) percentage greenspace based on Department of Communities and Local Government data produced from an enhanced basemap [22].

Data was accessed via the Greater London Authority Datastore LSOA Atlas [22] and the Nomis website [28].

Hospital of admission was identified as a potential confounder as it may be associated with the outcome due to different admission or coding policies and with the exposures of interest as the hospitals have different, although overlapping, catchment areas. Information on the hospital to which each admission occurred was available. Hospital of admission was coded as one of the four general hospitals with Emergency Departments that principally serve the area: King’s College Hospital (KCH), St Thomas’ Hospital (STH), University Hospital Lewisham (UHL) or Croydon University Hospital (CUH), or Other. The hospitals within the study area have overlapping catchments. To determine the population denominator when calculating admission rates for each hospital, the populations of LSOAs with admissions to more than one hospital were split between the hospitals according to the proportion of total self-harm admissions for the LSOA that attended each hospital.

### Statistical analyses

Unadjusted, unsmoothed rate ratios and observed/expected ratios standardised for 5-year age group, sex and calendar year, were first fitted using a Poisson regression model at LSOA level for the association of each exposure of interest with self-harm first admission rates. Standardised admission rate ratios (SARs) were calculated for each LSOA, with an SAR of 1 indicating self-harm admission rates equal to the average for the area as a whole. SARs were smoothed using a Besag–York–Mollie (BYM) Bayesian disease mapping model [29], which includes separate spatially structured and unstructured area-level random effects, to account for over-dispersion and spatial structure. Smoothing reduces the influence of random noise given the low counts in individual areas and adjusts estimates for spatial auto-correlation. The a priori confounders; hospital of admission, deprivation and social fragmentation, were added to the model individually and then together. The residual SARs after spatial smoothing and adjustment for confounders, which represent the remaining variation, were mapped to display spatial patterning.

Associations of the measures of urbanness and ethnicity with standardised self-harm first admission rates were then estimated, using a BYM model to control for the spatial structure of the data and adjusting for hospital of admission, deprivation and social fragmentation. Standardised rate ratios were estimated for the top versus the bottom quartile of each exposure with 95% credible intervals (CrI). A final model included all a priori confounders, population density, greenspace and ethnicity. The measure of proximity to the city centre was not included in this final model as it was very highly correlated with population density (correlation coefficient 0.84).

The overall degree of spatial variation in each model was quantified using two measures. The QR90 is the ratio of the SAR for the area at the 95th centile to the SAR for the area at the 5th centile and so describes the scale of variation in residual relative risk between the top and bottom 5% of areas. The spatial fraction estimates the proportion of the residual variance remaining after each model has adjusted for included variables that is due to spatial variation in the data. It is calculated by expressing the residual variance from the spatially structured random effect in the BYM model as a proportion of total residual variance from both spatially structured and unstructured random effects. If spatial fraction is close to 1 spatial heterogeneity dominates if it is close to 0 unstructured heterogeneity dominates.

The stability of spatial patterns over time were checked by running spatially smoothed models on data with individuals’ first admission each year for 2007–2009, 2010–2012 and 2013–2016 separately. A model smoothing for time as well as space using a random walk of 1 was run on this data for the whole period, using a “mixture model” to examine evidence of meaningful space–time interactions [30].

Analyses were carried out in R version 3.2.2. Bayesian models were run using Markov Monte Carlo Chain (MCMC) simulation in OpenBUGS version 3.2.3 using the *R2OpenBUGs* routine. Results were mapped in ArcMap 10.6.

## Results

Over the 9.25-year period studied, there were 8327 first admissions to hospital for self-harm by individuals living within the study area. This increased to 9789 when individuals’ first admission each year was included for the calculation of rates by year. Table 2 shows rates and SARs for self-harm admission, standardised for age, sex and year of admission. Higher rates were seen in females, younger age groups and areas with greater deprivation and larger non-white ethnic-minority populations. However, proximity to the city centre, higher population density and less greenspace were all associated with lower rates of admission for self-harm, before and after standardisation.

**Table 2:** Unadjusted and age, sex and year standardised rates of first admission for self-harm in South East London, 2007–2016, by individual and lower super output area characteristics

	First admissions (%)	Person years (1000s)	Unadjusted rate/10,000 (95% CI)	Unadjusted RR (95% CI)	Standardised^†^ RR (95% CI)
Total	8327	9297	9.0 (8.8–9.2)		
Female				*p* < 0.0001^b^	
15–19	1244 (24.6)	317	39.3 (37.2–41.5)	1	
20–24	851 (16.9)	449	18.9 (17.7–20.3)	0.48 (0.44–0.53)	
25–34	1102 (21.8)	1216	9.1 (8.5–9.6)	0.23 (0.21–0.25)	
35–64	1648 (32.6)	2147	7.7 (7.3–8.1)	0.20 (0.18–0.21)	
65+	205 (4.1)	620	3.3 (2.9–3.8)	0.08 (0.07–0.10)	
Male				*p* < 0.0001^b^	
15–19	293 (8.9)	319	9.2 (8.2–10.3)	1	
20–24	459 (14.0)	413	11.1 (10.1–12.2)	1.21 (1.05–1.40)	
25–34	809 (24.7)	1206	6.7 (6.3–7.2)	0.73 (0.64–0.84)	
35–64	1555 (47.5)	2126	7.3 (7.0-7.7)	0.80 (0.70–0.90)	
65+	161 (4.9)	482	3.3 (2.9–3.9)	0.36 (0.30–0.44)	
Year of admission^‡^				*p* = 0.32^a^	
2007/2008	2029 (20.7)	1907	10.6 (10.2–11.1)	1	
2009/2010	2090 (21.4)	1959	10.7 (10.2–11.1)	1.00 (0.94–1.07)	
2011/2012	2106 (21.5)	2023	10.4 (10.0-10.9)	0.98 (0.92–1.04)	
2013/2014	2229 (22.8)	2079	10.7 (10.3–11.2)	1.01 (0.95–1.07)	
2015/2016	1335 (13.6)	1330	10.0 (9.5–10.6)	0.94 (0.88–1.01)	
Hospital				*p* < 0.0001^a^	*p* < 0.0001^a^
KCH	1398 (16.8)	2049	6.8 (6.5–7.2)	1	1
UHL	1911 (22.9)	1850	10.3 (9.9–10.8)	1.51 (1.41–1.62)	1.52 (1.42–1.63)
CUH	2544 (30.6)	2320	11.0 (10.5–11.4)	1.61 (1.51–1.72)	1.64 (1.54–1.75)
STH	1217 (14.6)	1603	7.6 (7.2–8.0)	1.11 (1.03–1.20)	1.08 (1.00-1.16)
Other	1257 (15.1)	1476	8.5 (8.1–9.0)	1.25 (1.16–1.35)	1.26 (1.17–1.36)
IMD				*p* < 0.0001^b^	*p* < 0.0001^b^
Lowest quartile	1492 (17.9)	2251	6.6 (6.3–7.0)	1	1
2	1898 (22.8)	2431	7.8 (7.5–8.2)	1.18 (1.10–1.26)	1.11 (1.04–1.19)
3	2349 (28.2)	2347	10.0 (9.6–10.4)	1.51 (1.42–1.61)	1.42 (1.33–1.51)
Highest quartile	2588 (31.1)	2268	11.4 (11.0-11.9)	1.72 (1.62–1.83)	1.58 (1.49–1.69)
Social fragmentation				*p* = 0.002^b^	*p* < 0.0001^b^
Lowest quartile	2011 (24.2)	2122	9.5 (9.1–9.9)	1	1
2	1976 (23.7)	2188	9.0 (8.6–9.4)	0.95 (0.90–1.01)	0.93 (0.87–0.99)
3	2061 (24.8)	2353	8.8 (8.4–9.1)	0.92 (0.87–0.98)	0.90 (0.85–0.96)
Highest quartile	2279 (27.4)	2633	8.7 (8.3–9.0)	0.91 (0.86–0.97)	0.87 (0.82–0.93)
Measures of urbanness					
Distance				*p* < 0.0001^b^	*p* < 0.0001^b^
Furthest from centre	2261 (27.2)	2185	10.3 (9.9–10.8)	1	1
2	2323 (27.9)	2284	10.2 (9.8–10.6)	0.98 (0.93–1.04)	0.95 (0.89–1.01)
3	1983 (23.8)	2372	8.4 (8.0-8.7)	0.81 (0.76–0.86)	0.74 (0.70–0.79)
Closest to centre	1760 (21.1)	2455	7.2 (6.8–7.5)	0.69 (0.65–0.74)	0.55 (0.52–0.60)
Population density			*p* < 0.0001^b^	*p* < 0.0001^b^	
Lowest quartile	2578 (31.0)	2184	11.8 (11.4–12.3)	1	1
2	2242 (26.9)	2233	10.0 (9.6–10.5)	0.85 (0.80–0.90)	0.84 (0.80–0.89)
3	1719 (20.6)	2413	7.1 (6.8–7.5)	0.60 (0.57–0.64)	0.59 (0.56–0.63)
Highest quartile	1788 (21.5)	2466	7.2 (6.9–7.6)	0.61 (0.58–0.65)	0.58 (0.54–0.61)
% Greenspace				*p* < 0.0001^b^	*p* < 0.0001^b^
Most greenspace	2090 (25.1)	2189	9.5 (9.1–10.0)	1	1
2	2128 (25.6)	2322	9.2 (8.8–9.6)	0.96 (0.90–1.02)	0.95 (0.89–1.01)
3	2171 (26.1)	2381	9.1 (8.7–9.5)	0.95 (0.90–1.01)	0.94 (0.89-1.00)
Least greenspace	1938 (23.3)	2405	8.1 (7.7–8.4)	0.84 (0.79–0.90)	0.84 (0.79–0.90)
% Non-white ethnicity			*p* < 0.0001^b^	*p* < 0.0001^b^	
Lowest quartile	1646 (19.8)	2341	7.0 (6.7–7.4)	1	1
2	2152 (25.8)	2407	8.9 (8.6–9.3)	1.27 (1.19–1.36)	1.23 (1.15–1.31)
3	2388 (28.7)	2353	10.2 (9.8–10.6)	1.44 (1.36–1.54)	1.35 (1.27–1.43)
Highest quartile	2141 (25.7)	2196	9.7 (9.3–10.2)	1.39 (1.30–1.48)	1.27 (1.19–1.35)

*KCH* King’s College Hospital, *STH* St Thomas’ Hospital, *UHL* University Hospital Lewisham, *CUH* Croydon University Hospital

^†^Standardised for sex, 5-year age band and year of admission, ^‡^based on individuals’ first admission each year, ^a^likelihood ratio test, ^b^likelihood ratio test for trend

**Fig. 2:**
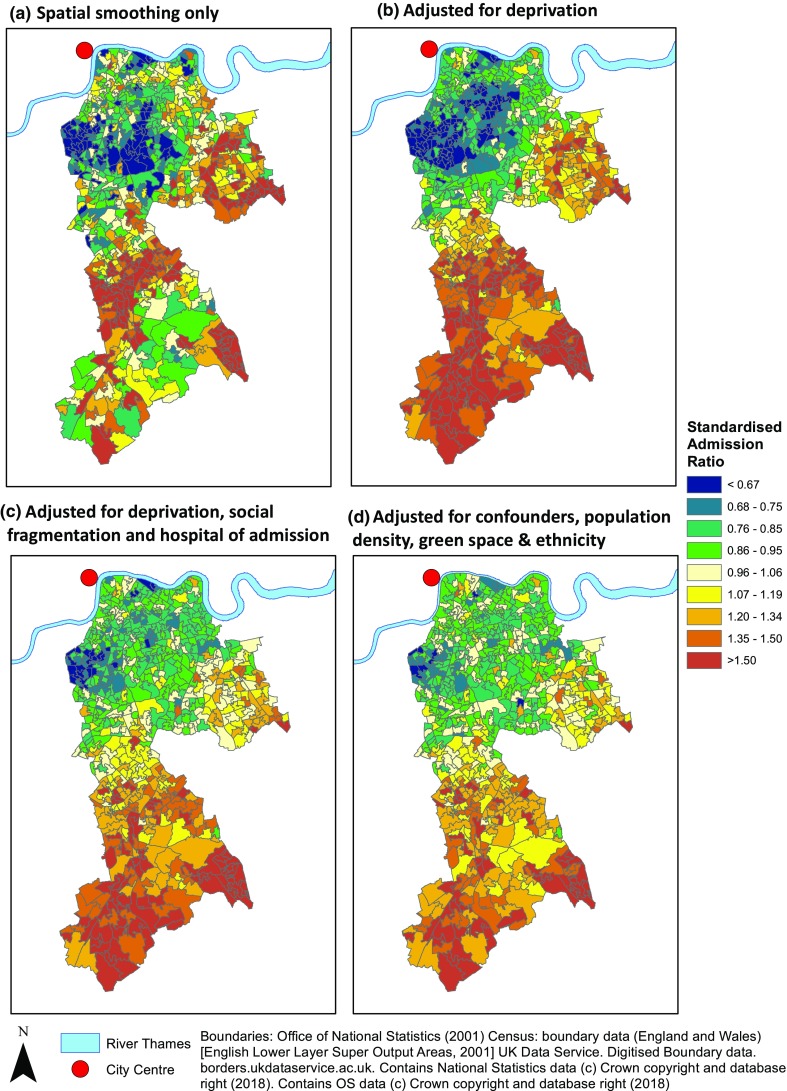
Residual age, sex and year standardised admission rate ratios for self-harm by lower super output area after spatial smoothing and adjustment

Figure 2 shows maps of SARs by LSOA. When smoothed and adjusted for spatial structure, a pattern was visible with clusters of areas with rates of self-harm admission lower than the area average in the north-west, closer to the city centre, and areas with higher than average rates further from the city centre in the south and east. Overall there was substantial variation between areas with a QR90 of 3.99 (credible interval, CrI 3.61–4.39), meaning a fourfold difference in self-harm admission rate between areas at the 95th centile compared to the 5th centile.

Adjusting for deprivation made the spatial pattern clearer and increased the spatial fraction from 0.67 (CrI 0.55–0.80) to 0.82 (CrI 0.73–0.91). Almost all the areas with lower than average rates of admission were found close to the city centre while higher than average rates were found further out. Adding the other a priori confounders, hospital of admission and social fragmentation, reduced the effect sizes but did not alter the spatial pattern seen.

Table 3 shows associations between measures of urbanness and ethnicity and SARs for self-harm. When effects were only adjusted for spatial structure, lower rates of self-harm admission were associated with both proximity to the city centre (closest versus furthest quartile SAR 0.67, CrI 0.48–0.89) and less greenspace (0.90, 0.82–0.99), indicating rates were lower in more urban areas. Higher ethnic-minority populations were associated with higher rates of self-harm (1.39, CrI 1.23–1.57).

**Table 3:** Association of measures of urbanness with lower super output area admission rates for self-harm

	Standardised^a,b^ RR (CrI)	Standardised^a^ adjusted^c^ RR (CrI)	Standardised mutually adjusted^d^ RR (CrI)	Standardised mutually adjusted^e^ RR (CrI)
Proximity				
Most versus least central quartile	0.67 (0.48–0.89)	0.71 (0.54–0.96)		
Population density				
Most versus least dense quartile	1.15 (0.94–1.39)	0.89 (0.76–1.06)	0.90 (0.76–1.05)	0.92 (0.77–1.07)
% Greenspace				
Least versus most quartile	0.90 (0.82–0.99)	0.99 (0.91–1.08)	1.00 (0.92–1.09)	0.99 (0.91–1.08)
% Non-white ethnic minorities				
Highest versus lowest quartile	1.39 (1.23–1.57)	0.93 (0.82–1.05)		0.93 (0.81–1.04)

*RR* rate ratio, *CrI* 95% Credible interval

^a^Standardised for age, sex and year of admission

^b^Adjusted for spatial structure

^c^Adjusted for confounders (hospital of admission, deprivation and social fragmentation) and spatial structure

^d^Adjusted for confounders, urbanness measures and spatial structure

^e^Adjusted for confounders, urbanness measures, ethnicity and spatial structure

Proximity to the city centre remained associated with lower rates of self-harm after adjustment for deprivation, social fragmentation and hospital of admission, 0.71 (0.54–0.96) mirroring the spatial patterning seen in Fig. 2c. However, there was no evidence of an association between population density, greenspace or percentage non-white ethnic minorities after adjustment for these confounders when examined individually or simultaneously (all credible intervals crossing one). This was reflected in the minimal change in the spatial pattern of residuals (Fig. 2d) and in the spatial fraction (0.63, CrI 0.39–0.83 compared to 0.64, 0.44–0.83) seen with the addition of these factors to the model adjusted for confounders only. The direction of effect seen for ethnic-minority populations reversed after control for confounders; suggesting that larger ethnic-minority populations may be associated with lower rates of admission for self-harm (SAR 0.93, 0.82–1.05).

Sensitivity analyses showed that spatial patterns in each time period were similar before and after adjustment for deprivation (Supplementary Figure 2), except for a suggestion that SARs fell in the Lewisham, in the east of the study area, in the final time period. Models with and without temporal smoothing produced very similar results and there was no evidence of space–time interactions (data not shown).

## Discussion

We have shown that rates of admission for self-harm are highly spatially patterned at small area-level within an urban study area. The spatial patterning seen was not explained by the known area-level associations with self-harm: deprivation and social fragmentation. We confirmed previous studies which have shown deprivation to be strongly associated with area rates of self-harm. However, strikingly, deprivation did not act as an explanation of spatial patterning, but was rather shown to be masking a spatial pattern. The pattern could not be explained by spatially different practices of admission.

Mapping demonstrates the pattern, with areas further from central London having higher rates of self-harm admission. This was especially clear after adjusting for deprivation, but even prior to adjustment, the most inner-city areas, which have high levels of deprivation and social fragmentation, did not have high rates of self-harm. This is also shown by the decreasing SAR with greater proximity to the city centre. It is notable that, after control for spatial autocorrelation, rates of self-harm did not appear to be associated with two measures commonly used to represent the urbanness in research: population density and greenspace. Hence, the importance of urbanness in terms of location within the city may have been missed by looking at associations alone without mapping.

The finding that proximity to central London is associated with lower rates of self-harm runs counter to much other research which has found higher rates of self-harm in more urban versus rural settings [8, 10, 11]. Our findings do not confirm hypotheses that stressors of urban living related to high density housing and lack of greenspace contribute to mental ill health: in spatially smoothed models there was little evidence either were associated with self-harm rates. This may be because the study is investigating degrees of urbanness within a city, rather than contrasting urban and rural areas. There is some support for this in the literature. One UK-wide study found evidence that the relationship between “rurality” and self-harm was non-linear with lower rates in both the most rural and most urban areas and higher rates in the suburbs [9]. It may also be that area-level associations with self-harm vary between different types of cities. For example, there is evidence that Dublin has lower rates of self-harm than other Irish cities despite being the most urbanised [10].

We hypothesised that the ethnicity of areas’ populations may explain urban spatial patterning of rates of self-harm admission, a suggestion raised by previous work on suicide rates [16], as historically ethnic-minority populations in the UK have been concentrated in inner-city areas particularly inner London [17]. There is some evidence that rates of self-harm differ by ethnicity [18], however previous literature does not make it clear which direction such an association might be expected to be in. Rates have been found to be higher in some non-white ethnic-minority groups [18], including South Asian [19] and Black Caribbean [20] women, compared to the white population in other UK settings. However, those who identify as Black Africans represent the largest minority ethnic group in the inner London boroughs of Lambeth and Southwark, and there is evidence that rates of suicidal ideation and self-harm are lower in this population [21]. We found that areas with high non-white ethnic-minority populations had higher than average rates of self-harm, but that this effect was lost, and may even reverse, once area deprivation was adjusted for. Adjustment for ethnic-minority populations made little change to the spatial pattern seen, suggesting differences in population ethnicity do not explain lower rates of self-harm in inner London.

The use of admissions data means that a possible explanation for patterning seen would be differences in the thresholds for admission in inner versus outer city hospitals. As our study had information on hospital of admission we could demonstrate significant differences in admission rate by hospital [SAR for CUH versus KCH 1.64 (1.54–1.75)], highlighting the importance of considering hospital effects when using routinely collected data. However, adjusting for hospital of admission did little to explain the spatial pattern seen.

### Strengths and limitations

The use of routine data provided almost universal coverage, allowing the study population to be the entire population of the study area, while longitudinal linkage of HES data [24] also allowed use of one admission per individual, preventing bias due to a few individuals with multiple attendances. However, admissions data for self-harm will only represent a small proportion of total self-harm in the population. Many individuals who self-harm in the community do not seek help at all [31], and only a minority of those attending emergency departments for self-harm will be admitted to hospital [32]. The findings of the study may represent the patterning of more severe self-harm requiring inpatient admission. However, admission rates could also be influenced by variations in the demands on and policies of services. This could result in acts of self-harm of the same severity being treated differently between hospitals and time periods. Attempts have been made to adjust for this by standardising for year of admission and including hospital of admission as a confounder, but residual confounding may remain.

The study hospitals’ catchments overlapped, hence the populations of some LSOAs had to be split between catchments when creating the denominator for calculating rates by hospital. The information available to do this required an assumption that *within each LSOA* presentations to hospitals occurred in the same proportions as admissions. This may have had the effect of diminishing the true size of hospital effects, suggesting the effect sizes we found may be an underestimate. Further work should assess hospital presentations with self-harm rather than just admissions.

The study area was restricted to a specific context: South East London. Despite limiting the analysis to four London boroughs there was great diversity—with some of the most and least deprived areas in the UK. Limiting to this area had benefits in ensuring access to mental health services and medical care after self-harm are fairly homogenous across the area and allowed us to adjust for the potential confounding effect of hospital of admission. However, it is not clear how far these findings can be generalised to other urban settings. The conclusion that previously researched factors associated with self-harm do not explain the spatial patterning seen makes a case that the epidemiology of self-harm in a large, diverse city like London may be at odds with that of its surrounding areas, a finding that may be applicable to similar cities internationally.

The small numbers of admissions per area meant that data had to be aggregated over a long time period. Sensitivity analyses suggested that patterns of self-harm did not change substantially over the study period, although there may have been falling rates in one area. This could reflect changes in deprivation levels in the area, not captured by the IMD based on data from early in the study period. The exposures of interest were measured at one time point but in reality may have been changing, introducing measurement error that could have reduced our ability to detect associations. The measures used will also not have perfectly captured the underlying concepts represented: the measure of ethnicity used was crude and does not reflect the diversity within ethnic-minority populations in the area, while measures of deprivation and social fragmentation used, whilst well established in the literature, may not work well in the London context where elements within the composite measures such as private renting and housing costs have different distributions to other UK settings [17].

## Conclusions

This study suggests that there is substantial spatial variation in rates of self-harm admission within urban areas, with lower rates close to the city centre. This spatial patterning is driven by additional factors to those previously researched and not explained by ethnicity. While nationally self-harm rates may be higher in urban and deprived areas, this cannot be extrapolated to mean that within a city the inner-city is the highest risk area nor that risk will be principally patterned according to deprivation.

## Electronic supplementary material

Below is the link to the electronic supplementary material.

Supplementary material 1 (PDF 1514 KB)

Supplementary material 2 (PDF 1692 KB)
