# Supplementary material for: Spatial patterning of self-harm rates within urban areas
Source: Soc Psychiatry Psychiatr Epidemiol. 2018 Sep 26;54(1):69–79. doi: 10.1007/s00127-018-1601-3 (PMC6336929; doi:10.1007/s00127-018-1601-3)
Supplement: Supplementary file 1 — Supplementary material 1 (PDF 1514 KB) [file 127_2018_1601_MOESM1_ESM.pdf]

Figure S1. Distribution of deprivation, population density, green space and non-white ethnic minority populations across the study area by lower super output area.

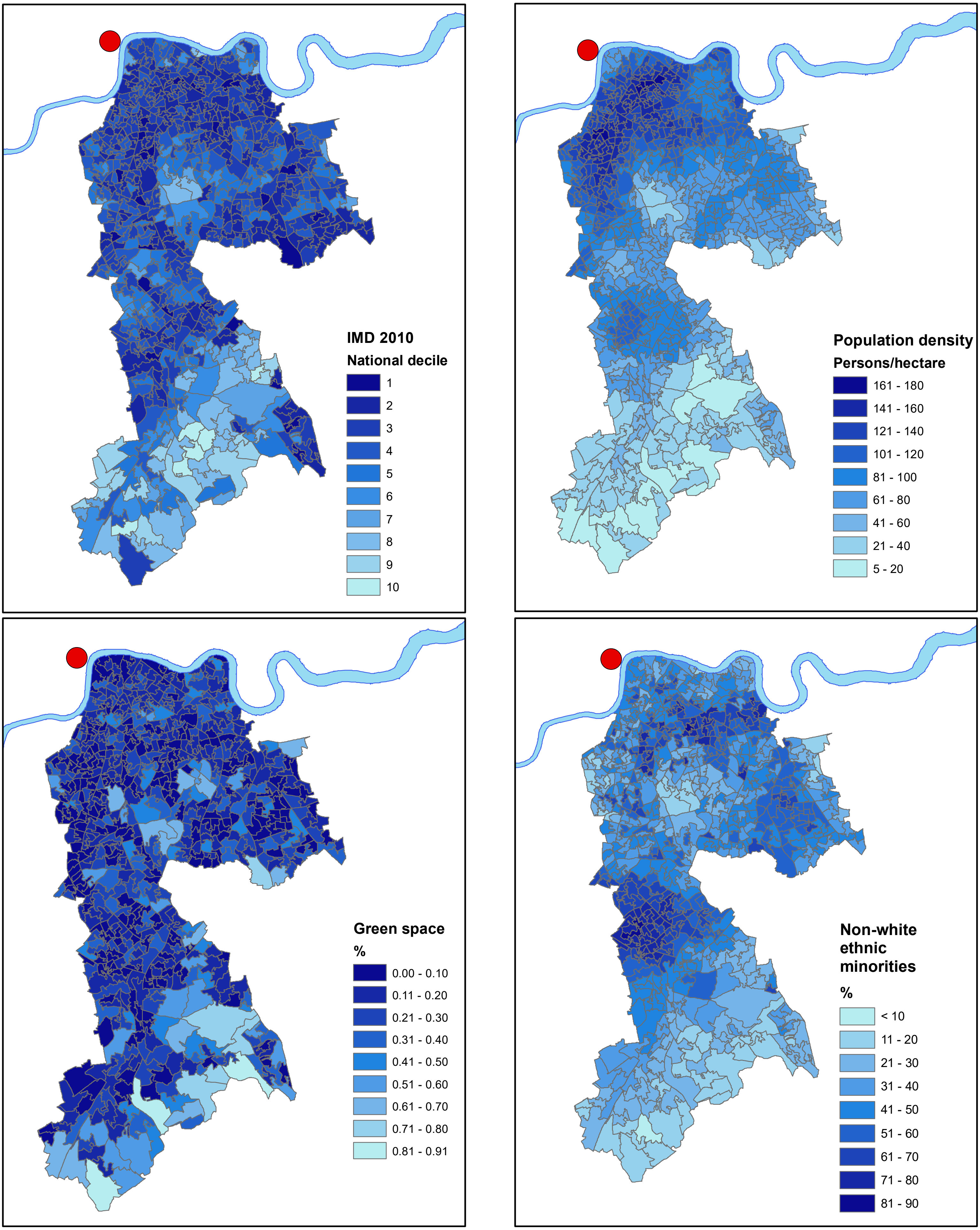

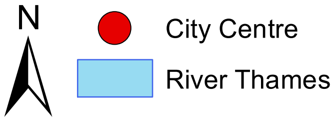

Boundaries: Office of National Statistics (2001) Census: boundary data (England and Wales) [English Lower Layer Super Output Areas, 2001] UK Data Service. Digitised Boundary data. borders.ukdataservice.ac.uk. Contains National Statistics data (c) Crown copyright and database right (2018). Contains OS data (c) Crown copyright and database right (2018)
Data: Indices of Multiple Deprivation, 2010. Department of Communities and Local Government
Other data, Census 2011, Office of National Statistics
