# Supplementary material for: Spatial patterning of self-harm rates within urban areas
Source: Soc Psychiatry Psychiatr Epidemiol. 2018 Sep 26;54(1):69–79. doi: 10.1007/s00127-018-1601-3 (PMC6336929; doi:10.1007/s00127-018-1601-3)
Supplement: Supplementary file 2 — Supplementary material 2 (PDF 1692 KB) [file 127_2018_1601_MOESM2_ESM.pdf]

Figure S2. Residual age and sex standardised admission ratios for self-harm by lower super output area after a) spatial smoothing and b) adjustment for deprivation, during three time periods*

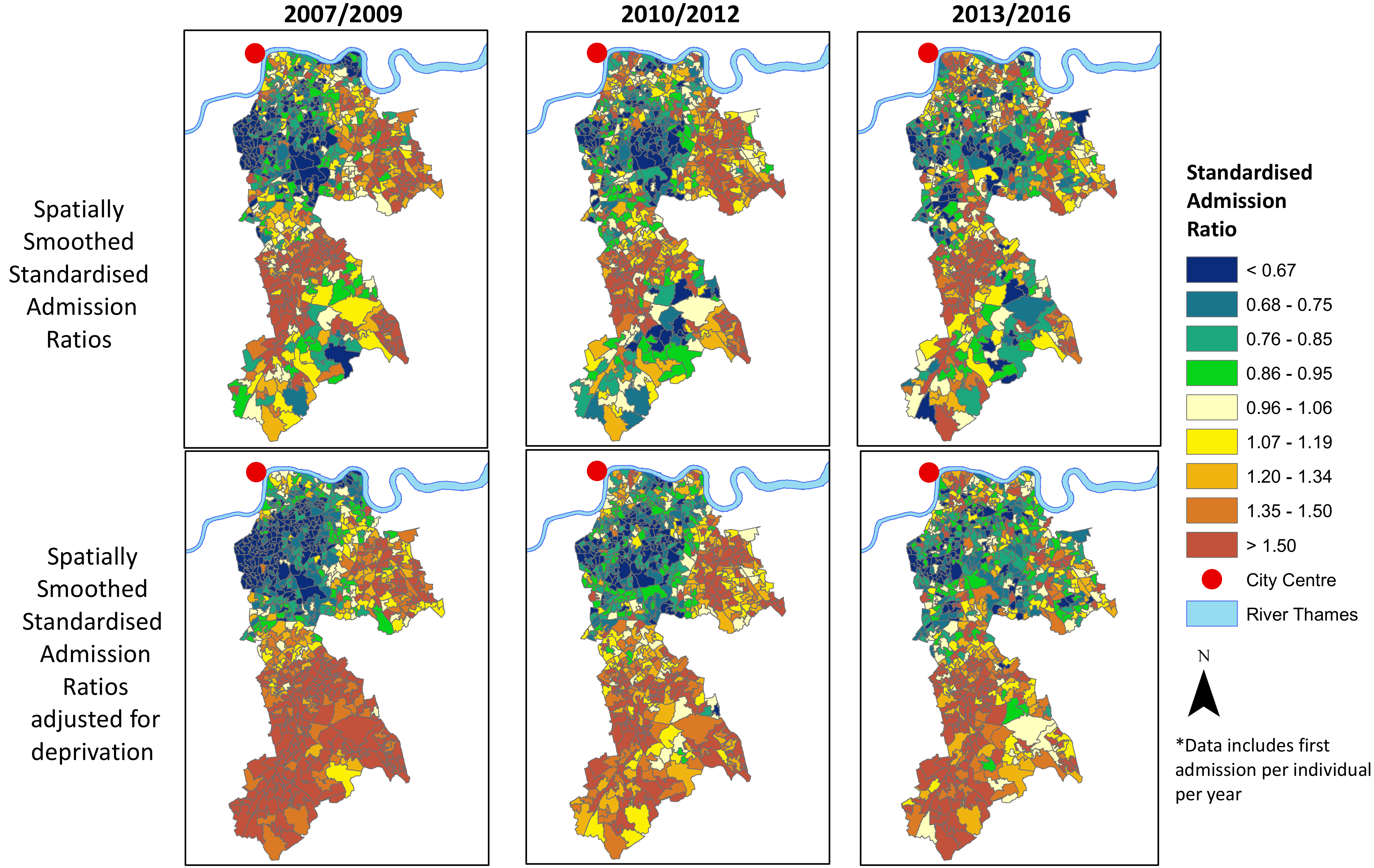

Boundaries: Office of National Statistics (2001) Census: boundary data (England and Wales) [English Lower Layer Super Output Areas, 2001] UK Data Service. Digitised Boundary data. borders.ukdataservice.ac.uk. Contains National Statistics data (c) Crown copyright and database right (2018). Contains OS data (c) Crown copyright and database right (2018)
